# The Insulin-Like Growth Factor 2 mRNA Binding Protein IMP2/IGF2BP2 is Overexpressed and Correlates with Poor Survival in Pancreatic Cancer

**DOI:** 10.3390/ijms20133204

**Published:** 2019-06-29

**Authors:** Charlotte Dahlem, Ahmad Barghash, Philip Puchas, Johannes Haybaeck, Sonja M. Kessler

**Affiliations:** 1 Department of Pharmacy, Pharmaceutical Biology, Saarland University, 66123 Saarbrücken, Germany; 2 Department of Computer Science, German Jordanian University, Amman 11180, Jordan; 3 Institute of Pathology, Medical University of Graz, 8010 Graz, Austria; 4 Department of Pathology, Medical Faculty, Otto von Guericke University Magdeburg, 39120 Magdeburg, Germany; 5 Department of Pathology, Neuropathology and Molecular Pathology, Medical University of Innsbruck, 6020 Innsbruck, Austria

**Keywords:** p62, RBP

## Abstract

The insulin-like growth factor 2 (*IGF2*) mRNA binding protein IMP2 (IGF2BP2) is an oncogenic protein known to be overexpressed in different tumor types. Pancreatic cancer is a very lethal cancer that requires early diagnosis and new treatment options. The aim of our study was to investigate the role of IMP2 in the initiation and progression of pancreatic ductal adenocarcinoma (PDAC). *IMP2* was significantly overexpressed in a human precursor (PanIN) lesions suggesting IMP2 as a marker for early stages of PDAC. In a PDAC cohort of matched normal and tumor samples *IMP2* showed overexpression in tumor tissues compared with normal pancreatic tissue. Strict correlation analysis (threshold *R*^2^ > 0.75) revealed 22 genes highly positively and 9 genes highly negatively correlating with *IMP2*. Besides genes involved in the inhibition of apoptosis (*Bcl-XL*), especially factors involved in ubiquitination were strongly correlated with *IMP2* expression: *SMURF1* and *FBXO45*. Moreover, protein kinase C (PKC) signaling pathway was distinctly affected: *DXS1179E* encoding PKC iota, PKC substrate *PLEK2*, and inositol triphosphate receptor *IP3R3* were positively correlated with *IMP2* expression. Besides tumor initiation, IMP2 also seemed to have an impact on tumor progression. TGF-β treatment of Panc-1 pancreatic cancer cells to induce epithelial-mesenchymal transition (EMT) was accompanied by increased *IMP2* expression. EMT is important for cancer cells to gain migratory and invasive potential, which is essential for metastasis. Concordantly, circulating tumor cells showed higher *IMP2* levels as compared with normal tissue from tumor origin and with normal hematological cells. Accordingly, IMP2 protein levels correlated with poor survival. In conclusion, as IMP2 seems to promote tumor progression of PDAC, it might be an interesting diagnostic and prognostic marker as well as a novel target for the treatment of PDAC.

## 1. Introduction

Pancreatic adenocarcinoma is the seventh leading cause of cancer-related deaths worldwide [1]. Prognosis is poor and 5-year survival is only 9%. Most of the patients have advanced stage tumors at the time of diagnosis making tumor resection impossible. Insulin-like growth factor 2 (*IGF2*) mRNA binding proteins (IGF2BPs/IMPs) have been described to be oncogenic in several types of cancer including pancreatic cancer [2,3,4,5,6,7]. The IMP family member IMP3 has originally been identified and pancreatic cancer tissues [8] and studied in this cancer type in more detail compared to the other two IMPs [9,10,11,12]. In lung cancer IMP1 has been reported to increase Kras signaling [13], which is frequently altered in pancreatic cancer tissue. Recently, *IMP2* has been reported to be the most abundant of the three members of the IMP family in most cancer types including pancreatic ductal adenocarcinoma (PDAC) [5]. However, beside gene expression in pancreatic cancer samples of the TCGA data set little is known about its role in pancreatic cancer progression and its prognostic relevance.

A well-known precursor of PDAC is Pancreatic Intraepithelial Neoplasia (PanIN). PanIN lesions progress from intraepithelial to invasive PDAC. Early detection of PanINs would help to interfere with PanIN progression to PDAC. IMP2 has been shown to promote carcinogenesis in the liver and to worsen chronic liver disease as a risk factor for liver cancer development [7,14].

This study shows for the first time that IMP2 expression is linked to progression and poor survival in pancreatic cancer.

## 2. Results and Discussion

### 2.1. IMP2 Is Overexpressed in Precursor Lesions, PDAC and Linked to Lower Rate of Survival

**Figure 1:**
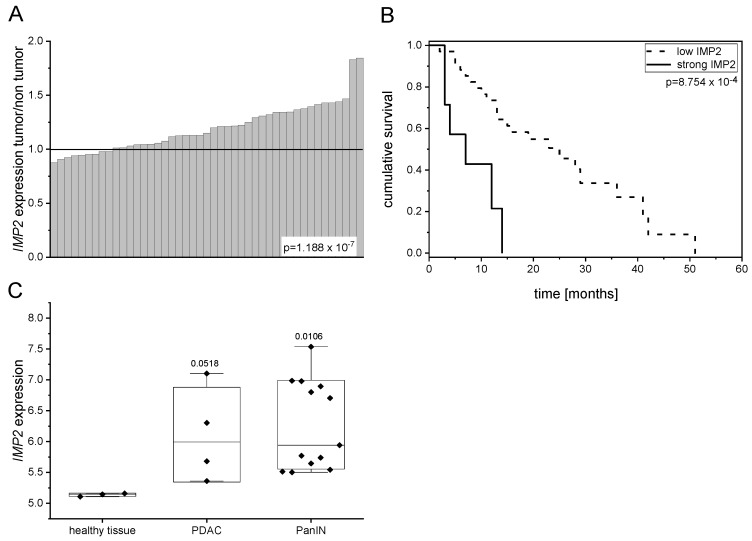
*IMP2* is overexpressed in PanINs and PDAC and leads to lower rate of survival. (**A**) Expression levels of *IMP2* in human PDAC cohort as compared with matched normal pancreatic tissue (GEO ID: GSE28735; *p* = 1.188 × 10^−7^; *n* = 45); (**B**) Kaplan-Meier estimated cumulative survival of PDAC patients with strong or low IMP2 expression (GEO ID: GSE28735; *p* = 8.754 × 10^−4^; low *IMP2* expression in tumor tissue < 6, *n* = 35; strong *IMP2* expression > 6, *n* = 7); (**C**) *IMP2* expression levels ±SEM in human PDAC and PanIN lesions (GEO ID: GSE43288).

In order to study the expression of *IMP2* in pancreatic cancer, publicly available datasets were investigated. Dai et al. recently showed that *IMP2* is overexpressed in PDAC tissues of the publicly available TCGA cohort compared to normal tissues [5]. In concordance, we observed *IMP2* overexpression in tumor tissues compared to normal tissues from a dataset containing matched normal and tumor samples (Figure 1A). Survival analysis revealed that high *IMP2* expression is linked to lower survival rate (Figure 1B). Interestingly, *IMP2* was overexpressed in PanIN lesions, which bear a high risk to develop pancreatic cancer (Figure 1C). In contrast to IMP2, IMP3 was shown to be highly specific for pancreatic tumor tissue and negative in premalignant tissues [15]. However, since biomarkers for early detection are needed to detect progression from PanIN towards PDAC, IMP2 might fulfill this need.

Strict correlation analysis (threshold *R*^2^ > 0.75) revealed 22 genes highly positively and 9 genes highly negatively correlating with *IMP2* (Table 1). Besides genes involved in the inhibition of apoptosis (*Bcl-XL*), especially factors involved in ubiquitination were strongly correlated with IMP2 expression: *SMURF1* and *FBXO45*. Moreover, protein kinase C (*PKC*) signaling pathway was distinctly affected: *DXS1179E* encoding PKC iota, PKC substrate *PLEK2*, and inositol triphosphate receptor *IP3R3*. Negatively correlated genes are involved in apoptosis regulation and DNA repair (*APO-J* and *CAF*) as well as epigenetic regulation (*AAM-B*). Interestingly, *IMP2* negatively correlated with *KIAA0922*, which antagonizes Wnt signaling, a pathway which has been described to be essential for pancreatic carcinogenesis [16,17].

### 2.2. IMP2 Is Involved in Metastasis

**Figure 2:**
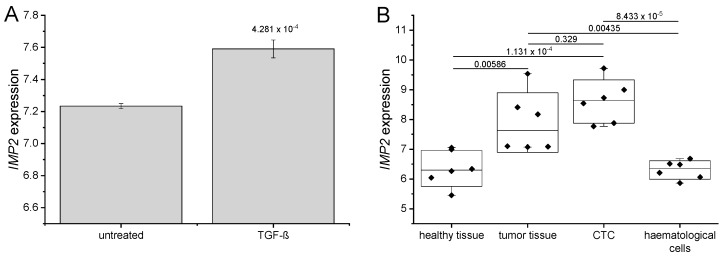
*IMP2* is associated with metastasis (**A**) IMP2 expression in Panc-1 cells after EMT induction by treatment with 5 ng/mL TGF beta for 48 h (GEO ID: GSE23952, *n* = 3); (**B**) *IMP2* expression in tumor tissue and CTC compared to healthy tissue and heamatological cells of the same donor as controls (GEO ID: GDS4329).

Epithelial-mesenchymal transition (EMT) is important for tumor cells to gain migratory and invasive potential. In glioblastoma, IMP2 promotes EMT and migration via the IGF2/PI3K/Akt pathway [18]. EMT can be induced in cell culture by treatment of cancer cells with TGF-β. In fact, TGF-β induced EMT was associated with increased *IMP2* expression (Figure 2A).

Metastases are a result of circulating tumor cells (CTC) that detach from the primary cancer and settle down in distant organs. In the publicly available dataset GDS4329 CTC, haematological cells, original tumour, and non-tumoural pancreatic control tissue were isolated from PDAC patients. CTC showed high *IMP2* expression, significantly increased compared to healthy pancreatic tissue as well as to haematological cells (Figure 2B), suggesting a role for IMP2 in metastasis of pancreatic tumors. IMP2 protein expression is linked to the occurrence of metastasis in esophageal cancer [3]. IMP2 was further described to be involved in tumor growth and metastasis in non-small cell lung cancer (NSCLC) and to be targeted by the tumor suppressive microRNA miR-485-5p [19]. Png and colleagues reported that IMP2 is secreted from metastatic cells and recruits endothelial cells during metastasis [20] underlining the role of IMP2 in tumor progression.

### 2.3. IMP2 Protein Is Overexpressed in PDAC Tissue Compared to Healthy Tissue and Associated with Lower Rate of One-Year Survival

**Figure 3:**
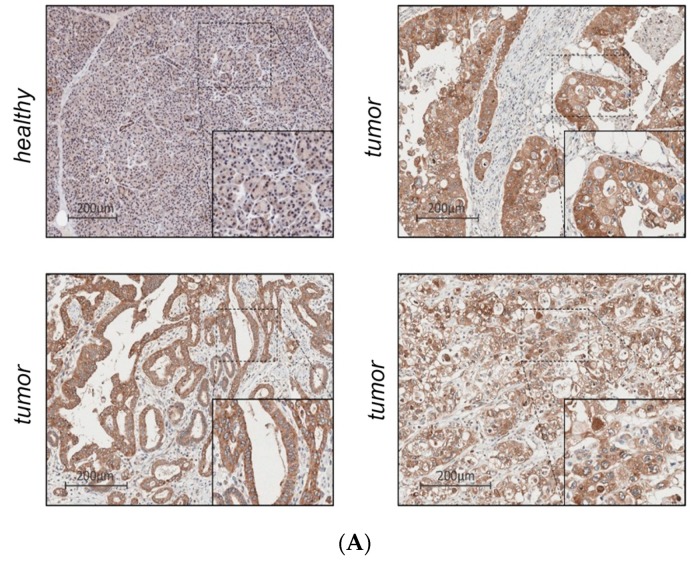

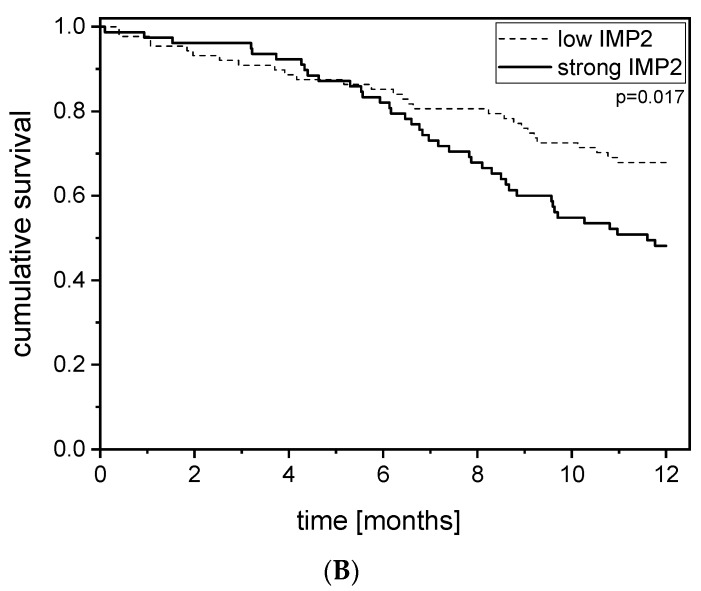
IMP2 protein is linked to poor one-year survival. (**A**) Tissue microarrays of PDAC sample collection from *n* = 210 PDAC patients (tumor tissue: *n* = 210 healthy tissue: *n* = 11) were analyzed by immunohistochemistry. IMP2 was significantly overexpressed in tumor tissue (*p* = 0.26 × 10^−4^). In healthy tissues IMP2 immunoreactivity was found in 91% of samples (score 0: 9%; score 1: 55%; score 2: 27%; score 3: 9%). All tumor tissues (*n* = 204) were positive for IMP2 expression: score 1: 7.4%; score 2: 40%; score 2/3: 2.9%; score 3: 49.5%); (**B**) Kaplan-Meier analysis of one-year survival of patients with strong IMP2 staining (score 3) versus low IMP2 staining in pancreatic tumor tissues.

Since increased protein levels are crucial for the usage of IMP2 as a biomarker, tissue microarrays of a PDAC sample collection from 210 PDAC patients in total were analyzed by immunohistochemistry. IMP2 was significantly overexpressed in tumor tissue (*p* = 0.26 × 10^−4^; Figure 3A). In healthy tissues IMP2 immunoreactivity was found in 91% of samples. (score 0: 9%; score 1: 55%; score 2: 27%; score 3: 9%). All tumor tissues (*n* = 204) were positive for IMP2: score 1: 7.4%; score 2: 40%; score 2/3: 2.9%; score 3: 49.5%). Kaplan-Meier analysis showed no effect of IMP2 staining intensity on overall survival, but strong IMP2 expression (score3) was linked to lower rate of one-year survival (Figure 3B). This is in accordance to findings in several other malignancies, in which a subgroup of tumors with highest IMP2 expression is linked to short survival [2,3,6,21,22,23].

In conclusion, IMP2 is frequently overexpressed in PDAC and significantly associated with poor prognosis. IMP2 seems to promote tumor progression of PDAC. Thus, it might be an interesting prognostic marker as well as a novel target for the treatment of PDAC.

## 3. Materials and Methods

### 3.1. Analysis of Human Gene Omnibus (GEO) Datasets

Preprocessed and normalized data from the RNA microarray GEO datasets GSE28735 [24,25], GSE43288 [26], and GDS4329 [27] were analyzed. In GSE28735 differential gene expression was analyzed between PDAC and non-tumor tissues (*n* = 45 each). Pearson correlation was applied to detect possible co-expressions between genes of interest and other genes in the dataset (threshold: *R*^2^ ≥ 0.75 or ≤−0.75, respectively).

### 3.2. Tissue Microarray and Immunohistochemistry

Formalin-fixed, paraffin-embedded pancreatic tissue samples and the corresponding clinical data were provided by the Biobank Graz under the permission of the ethics commission (Ethikkommission Medizinische Universität Graz, 12/2013, EK number 25-259 ex 12/13). A total of 200 patients (operated between 1991 and 2005) with a median age of 64 (range 31–81) years were retrospectively evaluated. The series included 184 ductal, 5 glandular, 3 intraductal papillary mucinous neoplasms (IPMN), and 2 endocrine tumors. For 25 patients survival data were missing. Immunohistochemical stainings against IMP2 were performed as previously described [23] using the Dako Envision AEC Kit (#K4009, Dako, Germany) for antibody detection according to the manufacturer’s instructions. TMAs contained three tissue spots per tumor. Stainings were evaluated for cytoplasmatic intensity by two independent, blinded investigators. Intensity was scored using the following scoring system: score 0 = no staining, score 1 = weak staining, score 2 = moderate staining, score 3 = strong staining. If the replicates of the same tumor differed in staining intensity median score was used for further analysis.

### 3.3. Statistical Analysis

Data analysis and statistics of experimental data were performed using either R software or OriginPro software (Origin 2019; OriginLabs). Differential expression analysis was based on the Kolmogorov–Smirnov test. Pearson correlation was applied to detect correlations between genes of interest. All tests are two-sided, and differences were considered statistically significant when *p*-values were less than 0.05. Data are shown as mean values ± SD (if not indicated differently), or as individual values and boxplots ± interquartile range with median. Depending on normal distribution, which was tested by the Shapiro-Wilk method, statistical differences were estimated by independent two-sample *t*-test or ANOVA (for multiple groups) combined with Tukey post hoc test, or Kruskal-Wallis-ANOVA respectively.

## Figures and Tables

**Table 1:** Genes correlating with *IMP2* expression. Table shows correlation coefficients for highly positively and negatively correlating genes (threshold *R^2^* > 0.75 or *R^2^* < −0.75, respectively).

Positive Correlation		Negative Correlation	
Gene	Correlation Coefficient *R*^2^	Gene	Correlation Coefficient *R*^2^
*ERO1-alpha*	0.867	*DMDL*	−0.833
*CD318*	0.830	***CAF***	−0.814
*ARVD12*	0.825	*SEPP1*	−0.801
*BEN*	0.818	***AAM-B***	−0.796
***BCL-XL/S***	0.793	*ADAMTSL3*	−0.779
***IP3R3***	0.787	*8B*	−0.774
*BM600-125KD*	0.783	***KIAA0922***	−0.765
***PLEK2***	0.781	*SEB*	−0.761
*TM9SF4*	0.776	*GGTA1*	−0.760
*DYT17*	0.774	***APO-J***	−0.753
*TMCC1*	0.772	*ADCL2*	−0.752
***DXS1179E***	0.770		
*HSNOV1*	0.764		
*SDC4*	0.762		
*TFGA*	0.761		
***SMURF1***	0.761		
*FAD104*	0.760		
*CT31*	0.759		
*FGD6*	0.758		
***FBXO45***	0.750		

## Abbreviations

IGF2	Insulin-like growth factor 2
IGF2BP2/IMP2	IGF2 mRNA binding protein IMP2
NSCLC	Non-small cell lung cancer
PDAC	Pancreatic ductal adenocarcinoma
CTC	Circulating tumor cells
